# Structural Sizing and Topology Optimization Based on Weight Minimization of a Variable Tapered Span-Morphing Wing for Aerodynamic Performance Improvements

**DOI:** 10.3390/biomimetics6040055

**Published:** 2021-09-26

**Authors:** Mohamed Elelwi, Ruxandra Mihaela Botez, Thien-My Dao

**Affiliations:** 1 Laboratory of Active Controls, Avionics and AeroServoElasticity LARCASE, ÉTS—École de Technologie Supérieure, 1100 Rue Notre-Dame Ouest, Montréal, QC H3C 1K3, Canada; mohamed.elelwi.1@ens.etsmtl.ca; 2 Research Laboratory in Machines, Dynamics, Structures and Processes, ÉTS—École de Technologie Supérieure, 1100 Rue Notre-Dame Ouest, Montréal, QC H3C 1K3, Canada; thien-my.dao@etsmtl.ca

**Keywords:** size and topology optimization, morphing variable span of tapered wing (MVSTW), aerodynamic optimization, OptiStruct solver, hypermesh

## Abstract

This article proposes the integration of structural sizing, topology, and aerodynamic optimization for a morphing variable span of tapered wing (MVSTW) with the aim to minimize its weight. In order to evaluate the feasibility of the morphing wing optimization, this work creates a numerical environment by incorporating simultaneous structural sizing and topology optimization based on its aerodynamic analysis. This novel approach is proposed for an MVSTW. A problem-specific optimization approach to determine the minimum weight structure of the wing components for its fixed and moving segments is firstly presented. The optimization was performed using the OptiStruct solver inside HyperMesh. This investigation seeks to minimize total structure compliance while maximizing stiffness in order to satisfy the structural integrity requirements of the MVSTW. The aerodynamic load distribution along the wingspan at full wingspan extension and maximum speed were considered in the optimization processes. The wing components were optimized for size and topology, and all of them were built from aluminum alloy 2024-T3. The optimization results show that weight savings of up to 51.2% and 55.7% were obtained for fixed and moving wing segments, respectively. Based on these results, the optimized variable-span morphing wing can perform certain flight missions perfectly without experiencing any mechanical failures.

## 1. Introduction

Aeronautical engineering research has made substantial progress in the past few decades due to the aviation industry requirements [1]. In global aerospace engineering centers, engineers and researchers have made extraordinary efforts to develop aircraft capable of adapting very well to various flight conditions [2,3]. The optimal aircraft performance capabilities refer to multiple missions’ realization under various flight conditions. The significant weight reduction obtained for robust and reliable structural aircraft configurations requires the integration of multidisciplinary design optimization approaches by use of a combination of advanced computer-aided design (CAD) with advanced computer-aided engineering (CAE) tools [4,5].

The aviation industry has invested in developing novel types of aircraft with very good performance, capable of meeting diverse flight requirements. These objectives have prompted engineers to develop new design techniques for reducing aircraft weight. Our structural optimization method to reduce aircraft weight needs to utilize topology, size, and shape optimization approaches. Hence, aircraft configurations are subjected to different constraints that must be addressed during the optimization [6,7]. The Topology optimization (TO) seeks to find the optimal distribution of materials across a certain design area under given constraints. Thus, it can determine an optimal structure by finding its optimal load, and therefore, its optimized material distribution. In the TO, when the material density of the design variables is equal to 1, these variables can be considered solid, which is critical for the structural design. On the other hand, when the material density is equal to 0, they can be considered void, thereby determining the best material distribution [8,9].

Multiple optimization techniques have been used in a variety of engineering design domains, and their application has spread throughout the field of aerospace engineering [10,11]. For example, several successful optimization techniques for finding the optimal structure of aircraft components are utilized in aircraft design, as seen in [12,13].

Airbus has applied Topology optimization in the A380 aircraft design program to generate its new lighter aircraft components. The most well-known optimized components for the Airbus A380 are the leading-edge ribs and the fuselage door intercostals, which led to weight savings of approximately 1000 kg for each aircraft [14]. The Boeing company took a similar approach when designing the wings’ leading-edge ribs for the B-787 Dreamliner. Topology optimization was incorporated with sizing and shape optimization to find the optimal wing leading-edge shape in the design process. As a result, the leading-edge ribs’ weight of the B-787 was reduced by 24–45% compared to the B-777 aircraft [15,16].

Oktay et al. [17,18] conducted research by combining the results from Computational Fluid Dynamics (CFD) analysis with the Computational Solid Mechanics (CSM) results obtained from Topology optimization. They investigated the aerodynamic load on wing lifting surfaces and used a Solid Isotropic Material with the Penalization (SIMP) topology optimization method to determine a wing’s optimal material distribution.

The Finite Element Method (FEM) and topology optimization (TO) have been linked with computer-aided engineering (CAE), and they are now considered the most advanced tools and methodologies in the aircraft design field. The combination of the FEM with TO makes it possible to obtain major weight reductions, thus resulting in material and fuel savings while maintaining the final product’s robustness properties [19,20]. Furthermore, optimization methods based on merging CFD with CSM allow the determination of the optimal wing shape, which could decrease the aircraft’s weight by obtaining its optimal aerodynamic performance [21].

The LARCASE laboratory at ÉTS presented a wide range of research activities in the multidisciplinary fields of aeroservoelasticity. Some of these studies were applied on unmanned aerial systems (UAS). The LARCASE team developed sophisticated methodologies for predicting the aerodynamic behavior and performance of the unmanned aerial systems UAS-S4 and UAS-S45 of Hydra Technologies [22,23,24]. This work concentrated on the structural analysis and optimization of unmanned aerial systems UAS-S4 and UAS-S45 based on CFD optimization results (aerodynamic performance, viscous damping and oscillations) [25,26]. The aerodynamic lift distribution over a wing’s entire surface was calculated using a numerical analysis based on the XFLR5 code results [27]. This wing was designed based on the variable-span morphing of the tapered wing (VSMTW) concept [28]. The optimization was implemented for the morphing wing, and it was developed based on the results of the preceding optimization process [10]. The topology optimization technique was used to determine the optimal positions of the wing components [29]. The optimization approach evaluated the efficiency of selecting candidate materials inside wing components, such as spars, ribs, and stringers, for high weight savings. Mechanical constraints impacted material characteristics, such as their overall strength, hardness, and robustness. TO established the optimal structural configuration with the highest reduction in its structural weight [30]. The optimized components of the wing must comply with the maximum rigidity of the structural wing configuration [31].

The first part of this investigation deals with allocating the best locations of wing components inside the VSMTW, based on the results of our TO methodology [29]. TO was applied based on the aerodynamic performance results obtained at the full wingspan extension and at the maximum speed (68 m/s); a safety factor and a 3 g load factor were considered. TO also suggested incorporating two spars, seven ribs, and several other support elements on the fixed and the moving segments with the aim to decrease the solid wing weights of fixed and moving segments for the optimized morphing wing from 112 kg to 16.3 kg, and from 45 kg to 10.3 kg, respectively. The Finite Element Analyses (FEA) were then executed, and their results indicated that the optimized VSMTW fulfilled the required mechanical properties such as linear elasticity and structural wing integrity. Thus, the optimized wing will withstand structural failure under extreme flight conditions.

## 2. Parametric Layout of the Optimized VSMTW

Global aerospace and aviation centers have conducted a high number of studies which demonstrated the wing shape modification benefits. Our initial investigation dealt with calculating wing aerodynamic performance by utilizing Computational Fluid Dynamics (CFD). That work proved that the wing area’s increase using its span morphing technique led to increased aerodynamic performance, with its associated fuel consumption savings and expanded flight envelope range [32]. Furthermore, instead of using conventional control surfaces, the asymmetric wingspan mechanism was used as a roll control system [28]. However, the main obstacle that design engineers must overcome is the structural wing weight penalty. Structural optimization is the most efficient method for reducing structural weight without compromising the wing’s structural integrity and its strength properties.

An optimized baseline wing was designed based on the TO results, and subsequently examined using Finite Element Analysis. Based on several test cases, this optimized wing demonstrated excellent mechanical behavior and reliable structural integrity for given boundary conditions [29].

### 2.1. Wing Design Configurations

**Figure 1:**
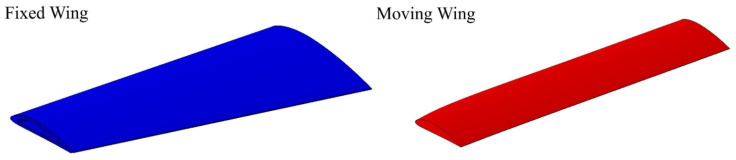
The initial geometrical shape of a variable-span morphing wing; fixed segment and moving segment.

A baseline wing was designed using the telescopic mechanism and the variable-span morphing wing theory based on the aerodynamic optimization results [28]. The wing was divided into two sections including moving and fixed segments, as shown in Figure 1. The CATIA V5 software was utilized to design the variable-span morphing wing.

**Figure 2:**
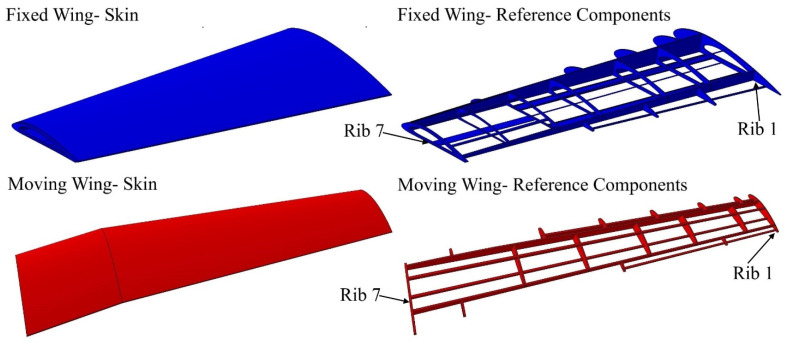
Geometrical model configuration of fixed and moving segments with their components, according to topology optimization.

The topology optimization method was performed in order to effectively allocate the internal structural elements inside both segments. The data and boundary conditions were computed at sea level altitude and maximum speed. Moreover, a safety factor of 1.5 and a 3 g load factor was added to the designed wing to overcome the various flight conditions. TO found that both segments must have two spars, with an optimal distance between them of 351.559 mm for the fixed segment, and 163.379 mm for the moving segment. TO also suggested seven ribs; the new configurations of spars and ribs is therefore illustrated in Figure 2.

The ribs are situated in both segments in accordance with the topology optimization results from analyzing solid wing segments at the proposed locations, as listed in Table 1:

### 2.2. Material Choice

The aluminum alloy 2024-T3 was selected for the first topology optimization process. This investigation chose the same material to continue the optimization process with the aim to reduce wing component weight. The aluminum alloy 2024-T3 is an isotropic material with good durability and mechanical properties combined with high strength and resistance to fatigue. This material is commonly employed in the design of aircraft components. The aluminum alloy 2024-T3 material properties are represented in Table 2.

## 3. Load Cases

**Figure 3:**
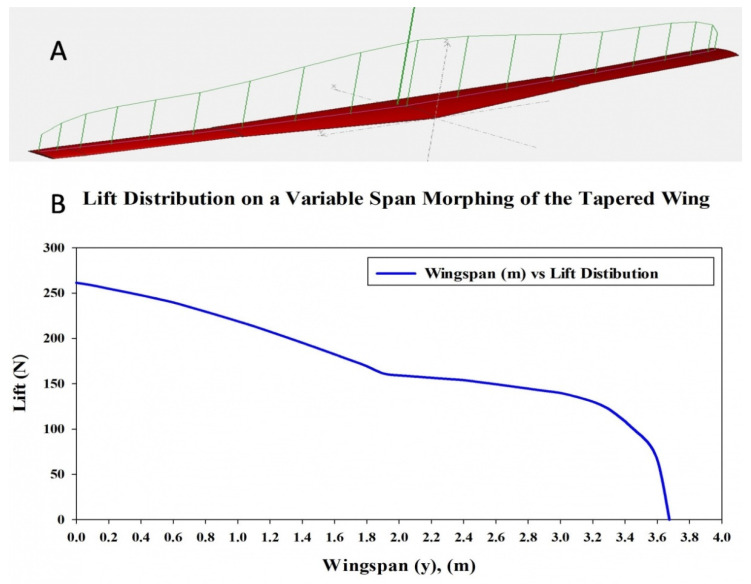
Lift distributions for the VSMTW along the wingspan by using (**A**) Fluent XFLR5 code, and (**B**) the chart of lift forces distribution along wingspan length.

This section assesses the lift distribution along the wingspan. First, the VSMTW was divided into sections with the aim to estimate the flow circulation distribution *Γ*_(*y*)_. For this reason, CFD Fluent code were combined with XFLR5 software results to evaluate the lift distribution along the VSMTW. Finally, the aerodynamic loads along the wing were measured using the lifting line theory, as illustrated in Figure 3.

The Kutta–Joukowski theorem was applied to estimate the flow circulation distribution along the span of the VSMTW by use of the following equation [33,34]:

(1) $$\Gamma_{\left(y\right)}=\Gamma_{0}{\left(1-{\left(\frac{2y}{b}\right)}^{2}\right)}^{1/2}$$

where $\Gamma_{\left(y\right)}$ is the flow circulation distribution calculated at any arbitrary location along the wingspan. $\Gamma_{\left(y\right)}$ is maximum when *y* = 0 and tends towards zero when *y* = ±b/2. Hence, the circulation at the wing mid-span $\Gamma_{0}$ can be obtained according to:

(2) $$\Gamma_{0}=\frac{4L}{\rho Vb\pi }$$

and thus, the lift distribution which acts on each wing segment was obtained using the following equation:

(3) $$L_{\left(y\right)}=\rho V\Gamma_{\left(y\right)}$$

**Figure 4:**
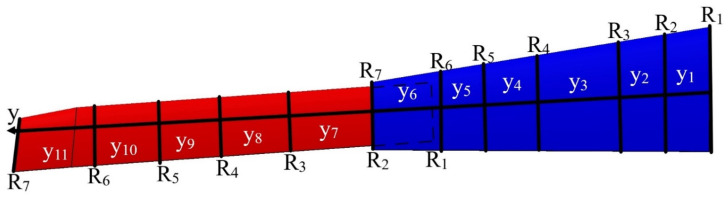
Generic planform wing with its span sections (*y_n_*) and chord numbers for the VSMTW.

After establishing the number and position of wing components for the VSMTW, the number of spars and ribs was determined based on the first topology optimization process. The Prandtl lifting-line theory was found to be the most efficient approach, as it allowed the lift to be distributed appropriately over the whole wingspan. The main parameters of the wingspan and its ribs were then determined. The first topology optimization suggested the use of seven ribs inside the moving wing segment and fixed wing segment at the proposed locations, as shown in Figure 2. The VSMTW was divided into 11 sections based on the rib locations at the wing full extension (75%) of its original length, as illustrated in Figure 4.

A safety factor of 1.5 and a 3 g load factor were also included in the modeling of the wing structural components. A safety factor of 1.5 was selected in accordance with the FAA regulations (FAR 25.303). According to these regulations, aircraft structures must withstand static loads, that were determined in this paper by their corresponding aerodynamic pressures without causing any structural damage or failure. The fixed wing ribs were evaluated using the average values of lift loads calculated for the fixed segment. The same approach was chosen for the moving segment, with its load’s values listed in Table 3.

## 4. Optimization Mathematical Model

The combination of sizing and topology optimization (STO) is a powerful approach for reducing structural configuration weight while maintaining its overall stiffness and structural integrity [35]. Furthermore, the sizing and topology optimization tools can be integrated into a multiple-computer-aided engineering software. In this investigation, STOP was performed based on the integration of the Finite Element Method (FEM) into an optimization solver.

This study’s main objective is the determination of the most efficient solution for producing lightweight components of a variable morphing wing while maintaining their optimal mechanical properties. Therefore, the STO results were obtained for ribs, spars, and support elements for each segment, and then were tested for the validation of their mechanical properties (displacements and stresses).

### 4.1. Topology Optimization Methodology

Topology optimization is a mathematical method used to formulate, and then to determine an optimum distribution of a material within a pre-defined design space. An optimized structural material configuration within a design domain can be obtained by considering its specific boundary conditions and constraints. The structural design fundamental materials are treated as solid, whereas null materials are regarded as void. Hence, the unnecessary structural weight can be eliminated. A commercial topology optimization application, Atair’s OptiStruct, is used in this investigation to manage the given design constraints by defining the objective functions for individual load cases. This application was designed to manage the constraints with the aim to ensure that design specifications were satisfied; therefore, in this paper, essential materials of the design are established, and the process to eliminate redundant elements is defined. Minimizing the compliance while maximizing the structural stiffness under specific design constraints can indeed meet the design requirements.

As mentioned above, the TO used in this study is combined with Finite Element Analysis (FEA), while the density-based approach is conducted. The design loads and parameters are applied to the wing components in order to deal with the material density distribution and its other mechanical properties (displacement, stresses, etc.).

TO is based on the Solid Isotropic Material with Penalization (SIMP) method. The design domain is optimized by minimizing the objective function, which refers to the structural compliance. The objective function variable is the material density, that defines whether a finite element is solid or void. The pseudo-density, *x_i_*, of the *ith* element may take values between 0 and 1, or *x_i_* (0 ≤  *x_i_*  ≤  1), wherein the 0 value represents a material as void, and the 1 value represents the material as solid. The pseudo-density variable can be defined with the following equation:

(4) $$x_{i}=\frac{\rho_{i}}{\rho_{0}}$$

where *ρ_i_* is the density of the *ith* element, *ρ*_0_ is the density of the base element, and *x_i_* is the pseudo-density of the *ith* element.

The formulation of the SIMP method defines the relationship between the material pseudo-density variable and its stiffness properties, as follows [36]:

(5) $$E_{\left(x_{i}\right)}=E_{solid}{\left(x_{i}\right)}^{p}$$

where $E_{solid}$ denotes the base material’s isotropic property and *p* is the exponent of the penalty parameter. Equation (5) can then be expanded the using the young modulus for solid and void regions:

(6) $$E_{\left(x_{i}\right)}=E_{void}+x_{i}{}^{p}\left(E_{solid}+E_{void}\right)\;\;\;\;\;\;\;\;\;\;\;\;p\geq 1$$

where $E_{void}$ is the low Young’s modulus assigned to void regions, $E_{solid}$ is the Young’s modulus assigned to solid regions, and $E_{\left(x_{i}\right)}$ is Young’s modulus assigned to each element.

The penalty factor value, *p*, should be selected to be large enough (*p ≥* 3 is generally considered), so that the volume constraint is active when the intermediate densities are penalized. Since the intermediate densities are close to 0, the material is unnecessary and may be therefore considered void. Contrarily, if the intermediate densities are close to 1, the material is critical to the structure’s integrity and can be considered as solid [37].

The volume fraction is a constraint to the total volume of the design domain related to the pseudo-density, as represented in the following equation:

(7) $$V=\overset{n}{\underset{i=1}{\sum }}x_{i}V_{i}$$

where *V* denotes the total volume and *V_i_* expresses the volume of the *ith* element.

The objective function of the TO is to minimize the structural compliance, that is subjected to the design constraints and can be defined with the following mathematical statement:

(8) $$\begin{array}{c} \underset{x}{\min}:c\left(x\right)=U^{T}KU=\sum_{e=1}^{N}{\left(x_{i}\right)}^{P}u_{e}^{T}k_{0}u_{e}\\ \mathrm{subject}\;\mathrm{to}:\frac{V\left(x\right)}{V_{0}}=f\\ \begin{array}{c} :KU=F\\ :0<x_{min}\leq x_{i}\leq 1 \end{array} \end{array}$$

in which the design variable vector, *x*, in the formulation *c*(*x*) presents the volume fraction $\frac{V\left(x\right)}{V_{0}}=f$, where *f* is the load vector, *V*_0_ is the initial volume, and *V* is the final volume. *K* is the global stiffness matrix and *U* is the global displacement, such that *KU* equals *F*, the force vector. In addition, *u_e_* represents the displacement vector, and *k*_0_ is the elemental stiffness matrix.

The shape of the density filter is selected as defined by Sigmund [38,39]. The filtration is defined for the physical relative density by $\tilde{x_{l}}$ and can be demonstrated using the following equation:

(9) $$\tilde{x_{l}}=\frac{\sum_{j\in N_{e}}w\left(r_{i}\right)v_{j}{\tilde{x}}_{j}}{\sum_{j\in N_{e}}w\left(r_{i}\right)v_{j}}$$

where $N_{e}=\left\{i|\|r_{i}-r_{e}\|\leq R\right\}$ denotes a neighborhood set defined by the filter radius, *R*. The filter radii around the centers of elements *i* and *e* are denoted by *r_i_* and *r_e_*, respectively. The weighting function is $w\left(r_{i},\;r_{e}\right)=R-\|r_{i}-r_{e}\|$, and the volume of the *ith* element is *v_i_* [38].

In this study, the major objective is to minimize each component’s weight within the optimized span morphing wing. TO ensures the lightest possible wing weight by maximizing its static stiffness. Therefore, minimizing compliance is a means to increase the structural stiffness subjected to the set load. The compliance optimization can be expressed as follows [37]:

(10) $$C=\overset{}{\underset{V}{\int }}fudV+\overset{}{\underset{S}{\int }}tudS+\overset{n}{\underset{i}{\sum }}F_{i}u_{i}$$

where *V* denotes the volume of the continuum, *f* is the distributed body force, *u* is the displacement area, *t* is the traction force, *Fi* represents the point load on the *ith* node, *u_i_* is the *ith* displacement degree of freedom, and *S* is the surface area of the continuum.

### 4.2. Formulation of the Stiffness and Topology Optimization Problem

The static stiffness can be obtained by applying static loading to wing components (for both segments). The deformation value under a static load refers to the displacement mechanical resistance [40]. The stiffness can be calculated based on the displacement, as follows:

(11) K(x)u=F

where *F* is the nodal forces vector, and *u* is the nodal displacement. The nodal displacement can then be found with the following equation:

(12) $$u\left(x\right)=K{\left(x\right)}^{-1}F$$

As already mentioned, the main objective of the structural TO of the morphing wing design problem is to maximize the wing’s stiffness while minimizing its weight. The density-based approach is coupled with the static linear method [41], so that the TO problem can be formulated mathematically as follows:

(13) $$\begin{array}{c} \min f\left(x\right)=f\left(x_{1},x_{2},\ldots \ldots ,x_{n}\right)\\ \begin{array}{cc} g_{j}\left(x\right)\leq 0, & j=1,\ldots \ldots ,m \end{array}\\ \begin{array}{cc} x_{i_{min}}\leq x_{i}\leq x_{i_{max}} & i=1,\ldots \ldots ,n \end{array} \end{array}$$

where *f*(*x*) represents the objective function, *g*(*x*) denotes the constraints, *x* are the design variables, which are considered as independent variables of the *f*(*x*), and *x_min_* and *x_max_* are the upper and lower bound constraints, respectively.

The TO algorithm coordinates the distribution of the material throughout the design domain by optimizing the user-defined objective function under certain constraints. In this study, each wing component’s design space for both segments was defined as a separate element in the optimization process.

The gradient-based optimization method is widely utilized, as it is recognized as a very efficient method. The constraints’ screening process was applied to compute the set constraints, thereby minimizing the required gradients.

The compliance of the static structural stiffness is completely transformed into potential deformation energy [42]. The static structural stiffness can be calculated using Finite Element Analysis (FEA). The TO solver OptiStruct uses algorithms to set up the optimal material distribution inside the design domain in order to achieve the optimal wing geometrical shape. Then, it computes the design structure that offers the optimum solution under given boundary conditions by minimizing the wing components’ compliance while respecting the design constraints to decrease their weights. The optimum solution is reached when the solution convergence is obtained in an iterative approach.

## 5. Wing Component Structure Design Optimization Process

This investigation’s primary goal is to design a robust structure by reducing the weight of a variable-span morphing tapered wing (VSMTW). The main challenge is to combine the strength with light weight properties of the wing. The objective is to maximize the stiffness by optimizing the material distribution within the wing components’ volume space. Many factors must be considered in the optimization process, including the computational area, mesh generation, and boundary condition settings. For more accurate and reliable results, the pressure was calculated separately for each wing component. The optimization settings of the wing components were found as described in the following sub-sections [43].

### 5.1. Wing Skin Thickness Size Optimization

**Figure 5:**
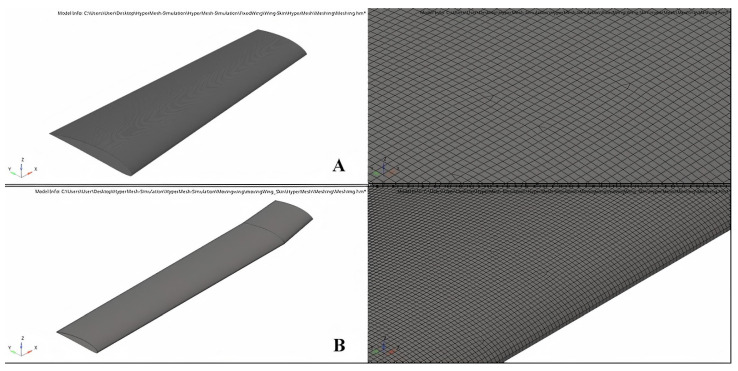
Finite Element for (**A**) fixed wing and (**B**) moving wing.

A baseline wing for both segments had a skin thickness of 2 mm for its optimization process. Size optimization (SO) was conducted in a 2D environment, and then the results were extended to a 3D environment, and they were further chosen to design the best possible wing skin [44]. In fact, it is known that SO is an iterative process. Wing skin optimization was performed using the aerodynamic loads calculated with the CFD solver and the XFLR5 code at the maximum speed and full wingspan extension. A 20 mm tetra quadrilateral element size meshing was applied on both segments, as shown in Figure 5.

The aerodynamic loads were applied, and the design variables were considered as scalar parameters. These parameters were defined for the optimization problem in this investigation because of the fact that the wing skin thickness influences the system responses. The optimization responses were defined for two variables, which were the design volume and the selected stress. The objective function concerned the minimization of the structural mass of the wing skin. The design constraints were determined in this investigation as the lower and upper bounds of the maximum stress values, which was less than 200 MPa due to the stress response based on the design variable. The design variable was defined with an initial value of 2 mm, with a lower bound of 0.5 mm and an upper bound of 2 mm [13,45]. The optimization problem was formulated as follows:

(14) $$\begin{array}{c} \min M\\ \sigma_{max}=200\;\mathrm{MPa}\\ 0.5\leq T\leq 2 \end{array}$$

### 5.2. Spar Structure Topology Optimization

**Figure 6:**
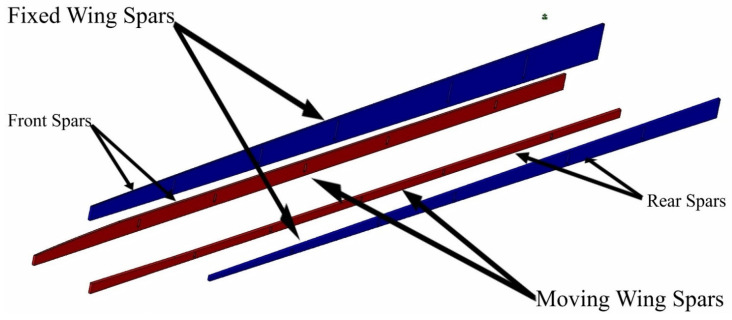
Baseline geometry shape for morphing wing spars for fixed and moving segments at their original position on the wing.

The spar is one of the most important wing components, as it can support the heaviest aerodynamic loads affecting the aircraft wing. A spar is designed as an extended beam along the wingspan, which supports the wing submitted to the bending load. Thus, it should withstand the impacts of shear, tensile, and compressive loads. In our project, the previous TO of baseline solid wings design suggested the use of two spars for each wing segment at defined locations, as shown in Figure 6 [29]. The aerodynamic loads were calculated with the ANSYS and XFLR5 software, where the spars were modeled as beams with discrete loads at different locations (their values are presented in Table 3).

The structural weight was considered in the problem formulation for the wing spars, as the main aim of our design was to find the optimal wing structural parameters. This objective was used to determine the wing minimum weight while satisfying its strength, durability, and versatility constraints [8].

The I-beam section was selected for modeling the wing spars (except at their connecting points with the ribs) because it provided high mechanical advantages compared to other beam section shapes. The I-beam shapes bear higher loads than the other beam section shapes, supported very well the other wing components, withstood mechanical fatigue (such as torsions and deformations), reduced the load intensity on the other wing components, and led to low costs and weight [46,47].

**Figure 7:**
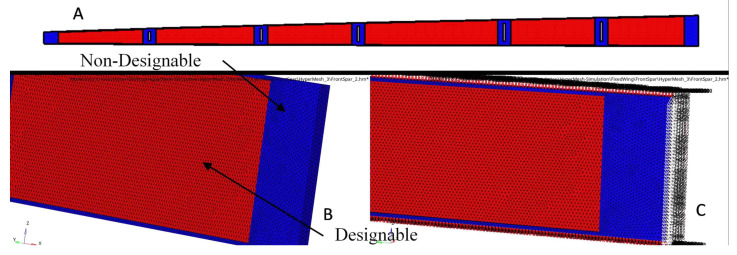
An example of the meshing and boundary conditions for the front spar—fixed wing. (**A**) Front spar for a fixed wing, (**B**) The meshing and the designable and non-designable areas of the front spar and (**C**) The boundary conditions of the front spar.

TO is an iterative process needed to reach the optimized system’s best-converged solution based on the objective function, and certain design constraints. In a 3D environment, all wing spars are subjected to the same analyses procedures [48]. As the selected loads are proportional to the wing spar length, the objective function includes both the designable region, as well as the flange and the attachment areas used to link the ribs inside the spars, which is considered a non-designable region. The non-designable regions were fixed during the optimization process as they were excluded from the design domain. Figure 7 illustrates the determined designable and non-designable regions of the front wing spar by creating various areas, and thus by leading to more feasible optimizations [49]. The spars meshed into uniform tetrahedrons with elements of 3 mm thickness; the uniform grids were needed to reduce the computation errors.

### 5.3. Rib Structure Topology Optimization

**Figure 8:**
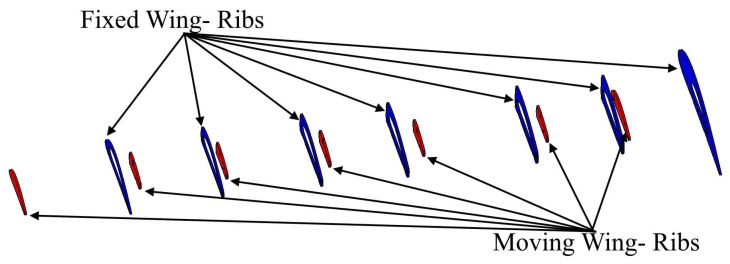
Baseline geometrical shape for morphing wing spars for fixed and moving segments at the original position.

The baseline TO proposes seven ribs for both wing segments, as shown in Figure 8, with approximate distances between ribs inside the wings [29]. These ribs are used to minimize the wing deformation by maintaining its segment’s geometrical structure. Thus, the external loads are evenly distributed on the wing skin. Fixed wing ribs should be designed with a cavity, as they house their moving segment, as illustrated in Figure 8. Therefore, the TO was implemented on the inner six ribs for both segments. The external ribs were not considered for the fixed and moving wing segments within the design optimization region, as the fixed wing’s last rib should only contain the cavity for the moving wing segment. Additionally, the ribs of the moving wing segment should have a closed geometrical shape.

The optimum material distribution inside the ribs was determined by the original design space configuration and the boundary conditions. A uniform tetrahedron mesh with elements of 3 mm in size was generated for the wing ribs for the two segments in order to implement TO. Both wing ribs were discretized into various elements, and their node numbers were based on their rib’s sizes.

**Figure 9:**
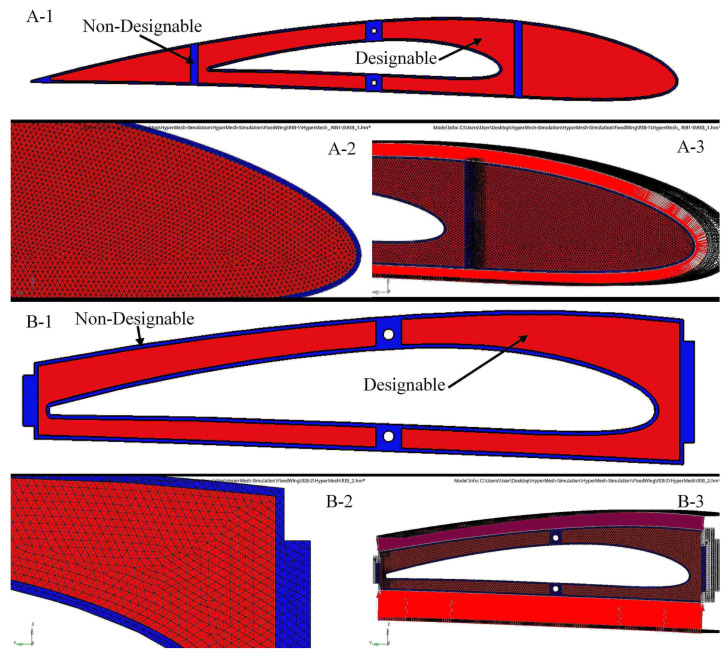
Geometry, meshing and boundary conditions for the ribs 1 and 2—fixed wing as an example. (**A-1**,**B-1**) Geometry for ribs 1 and 2 and the designable and non-designable areas for a fixed wing; (**A-2**,**B-2**) ribs 1–2 meshing; and (**A-3**,**B-3**) Boundary conditions of the ribs 1–2).

Figure 9 shows the meshing and its boundary conditions of the ribs for the fixed wing. A volume constraint was applied to all wing ribs in the optimization problem by its consideration as an opposite constraint. The aerodynamic analysis (see Table 3) can define the loads for each rib. The objective function was extended to the designable region and the rib edge. The link spots connecting the ribs inside the spars were considered as a non-designable region. The non-designable regions at the link spots with spars were stabilized during the optimization process, as they were excluded from the design domain.

### 5.4. Topology Optimization (TO) of the Support Element’s Structure

The initial design of the wing support elements, including its leading-edge ribs, trailing edge ribs, stringers, and stiffeners for the optimized wing based on the TO method were designed. The support elements of both wing segments were designed as shear webs to be stabilized against buckling loading, bending loading, torsion loading, and vertical shear loading. Other loads besides the aerodynamic loads and inertial loads applied to the wings can be considered in the design process according to their shapes and structural masses. In addition, the payload, expressed by electronic devices or surveillance cameras, can be carried on the wings and/or on the fuselage. This extra equipment adds more weight, thus contributing to the existing bending, shear, and torsion loads of the wing.

**Figure 10:**
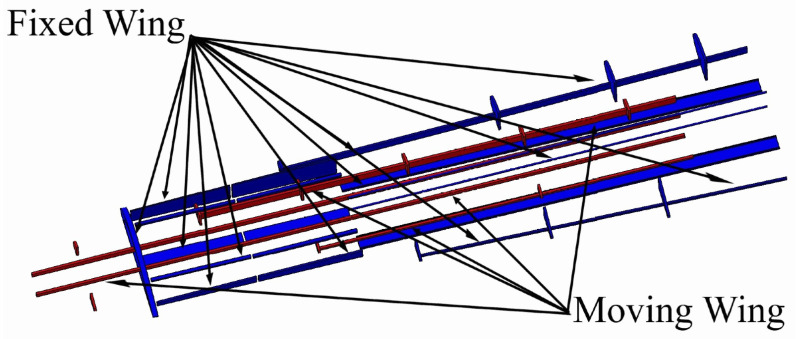
Support elements for fixed and moving wings at their original positions.

The shear web structure of wing components provides a reliable design by enabling them to carry loads on the wing in different directions. The support elements’ structure for both segments of a VSMTW is shown in Figure 10.

The current meshing and boundary conditions of support wing elements were similar to those of the spars and ribs. The layout of the support elements’ structure of the wing, the geometry of the mesh model, and its boundary conditions were established based on parametric data obtained from the aerodynamic investigation, as listed in Table 3. Since the support elements’ weight contributes significantly to the total weight of the wing, it is important to reduce it while maintaining the wing stiffness. The rigid meshes of the support elements for both segments were designed by applying the TO using uniform tetrahedrons with their element’s sizes of 3 mm.

**Figure 11:**
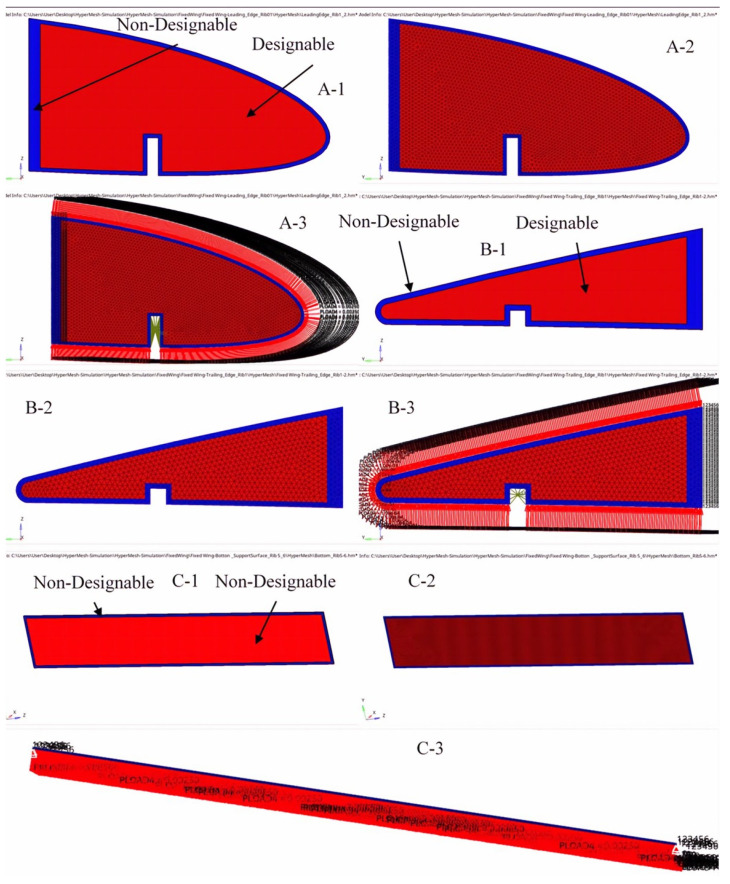
Examples of A—geometry, B—meshing and C—boundary condition for support elements (leading-edge rib 1, rib 2, and bottom support lamina between ribs 5 and 6). (**A-1**,**B-1**,**C-1**) Geometry for support elements and the designable and non-designable areas for a fixed wing; (**A-2**,**B-2**,**C-2**)-Support elements meshing; and (**A-3**,**B-3**,**C-3**) Boundary conditions of the Support elements).

**Figure 12:**
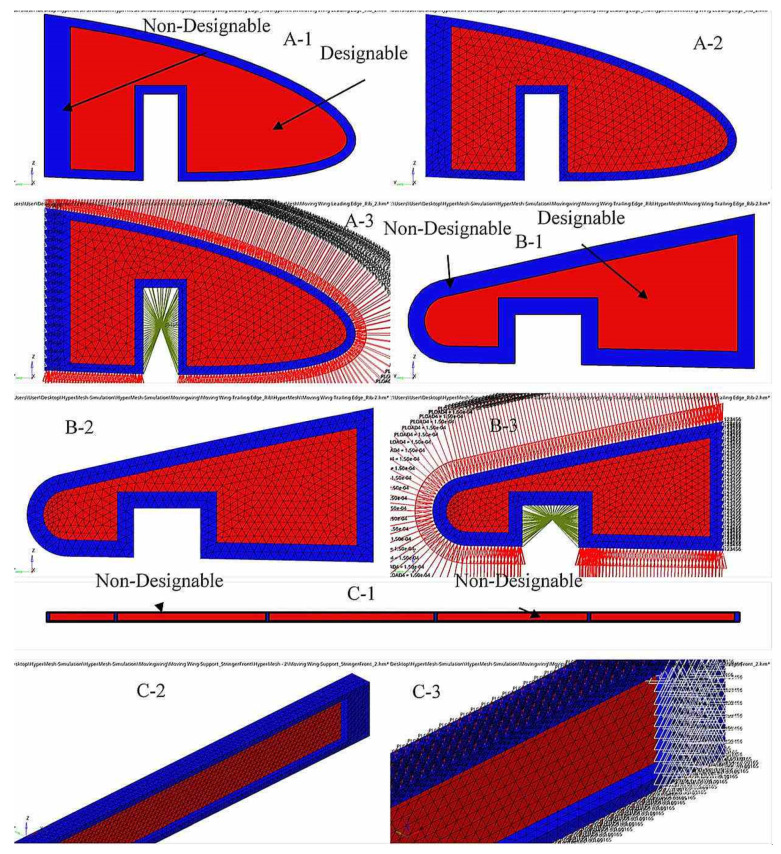
Examples of A—geometry, B—meshing and C—boundary conditions for support elements (leading-edge rib 1, trailing edge rib 2, and support stringer front). (**A-1**,**B-1**,**C-1**) Geometry for some support elements and the designable and non-designable areas for a moving wing; (**A-2**,**B-2**,**C-2**) support elements meshing; and (**A-3**,**B-3**,**C-3**) Boundary conditions of the support elements).

The number of meshing elements and nodes differs for each component, depending on its dimensions. Figure 11 and Figure 12 illustrate some support components’ meshing and boundary conditions for a fixed wing and a moving wing, respectively. A volume constraint was applied to the entire support element and served as an opposing constraint in the optimization problem.

The attached area between wing components cannot change from its initial design. Therefore, the edges of the support element components and the loci binding to other wing components were considered as non-designable areas. Since there are various places where links must be made to connect the wing components, such as spars and ribs, these components were defined with fixed areas during the TO. They represent the bonding with other wing components, and thus they can be altered throughout the optimization.

## 6. Results and Discussion

As described in the previous sections, aerodynamic analysis and optimization were conducted on the VSMTW for the UAS-S4 under different flight conditions [50]. Thus, their results were then integrated into the optimization problem. This optimization problem considered the parameters that characterized a structure’s overall performance.

**Figure 13:**
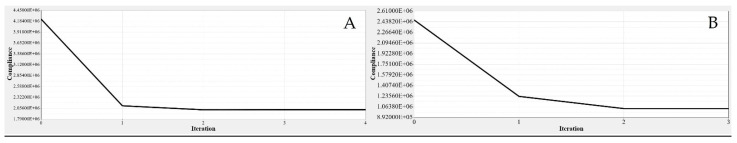
Convergence graph of the objective function for (**A**) fixed wing skin and (**B**) moving wing skin.

All these parameters were considered as either optimization objectives or constraints. Skin thickness and other wing components are known as sizing and topology variables, respectively. Sizing optimization (SO) of the wing skin for both segments was performed by adding the design constraints to the structural stiffness. A very small number of optimization iterations were used to converge rapidly towards the optimal solution by obtaining a highly efficient skin thickness, as shown in Figure 13.

**Figure 14:**
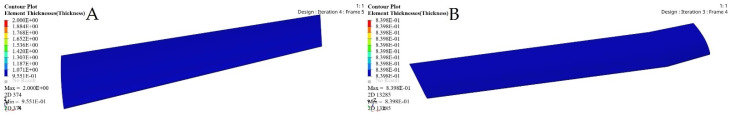
Sizing optimization (SO) results for (**A**) fixed wing and (**B**) moving wing.

SO was performed on both wing segments. The interpretations of its results for both wing segments are shown in Figure 14.

**Figure 15:**
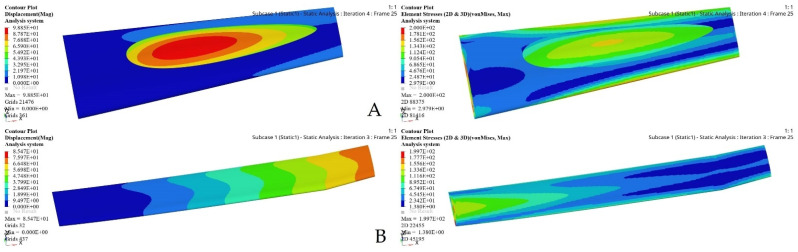
FEM results for fixed and moving wing segments: (**A**) fixed wing, and (**B**) moving wing, obtained using the sizing optimization problem.

An initial wing skin thickness was chosen to be 2 mm for both wing segments. The FEM evaluations of a fixed and a moving wing skin are shown in Figure 15. This optimization relies on an FEM static analysis under extreme flight conditions, and it creates stress and deformation.

The maximum stress value of the wing two segments skin was restricted to 200 MPa for the highest efficiency in order to withstand the aerodynamic loading acting on its surface. The same boundary condition was applied to both wing segments. Hence, SO and FEAs were based on the wing design volume and shape. The maximum deformations and stresses obtained under extreme flight conditions, as well as other important parameter values obtained from the optimization results, are shown in Table 4.

**Figure 16:**
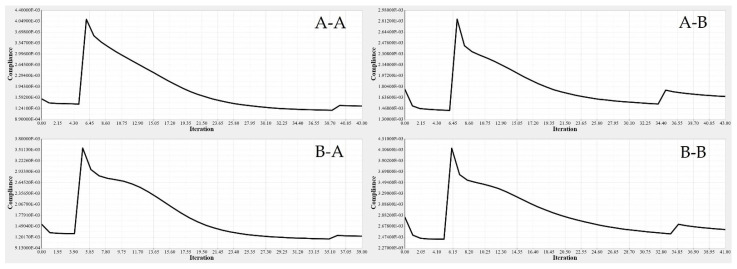
Convergence graph of the objective function: (**A-A**) front spars of a fixed wing, (**A-B**) rear spars of fixed wing, (**B-A**) front spars of a moving wing, and (**B-B**) rear spars of a moving wing.

TO was implemented to determine the spars that would achieve wing maximum stiffness and reduce the weight. A Computational Fluid Dynamics (CFD) study of the wing structure was conducted to obtain high levels of fidelity. This analysis provided the pressure loading cases for extreme flight conditions. The aerodynamic analysis of the wing structure was performed at the sea level altitude and maximum speed (68 m/s) to ensure the optimal distribution of aerodynamic loading over the wingspan, as shown in Table 3. The minimum structural compliance was obtained for the initial wing spars’ design for both segments by a various number of iterations based on the size and shape of each spar, as illustrated in Figure 16.

The minimization of structural compliance using constraints is defined as a volume fraction by 30%. The design variable is the structural weight for an allocated global compliance-based stress. The TO results are summarized in Table 5.

**Figure 17:**
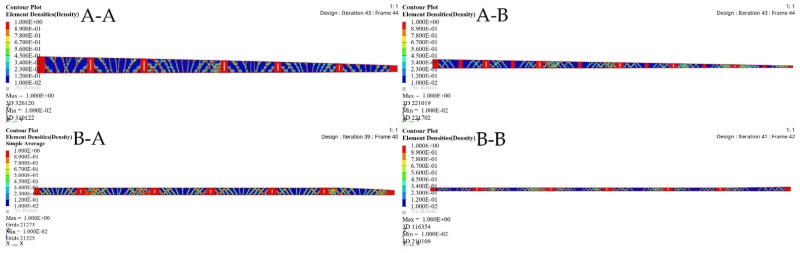
Element density variation with the numbers of iteration for (**A-A**) fixed wing front spars, (**A-B**) fixed wing rear spars, (**B-A**) moving wing front spars, and (**B-B**) moving wing rear spars.

TO based on the FE modeling of the wing spar is an iterative process, as shown in Table 5, and in Figure 16 and Figure 17. Table 5 shows the parameters of the most important wing spars by calculating them as a function of the design parameters.

**Figure 18:**
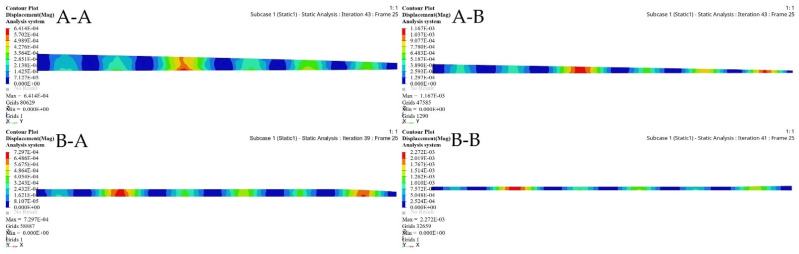
Deformation results based on topology optimization for (**A-A**) fixed wing front spars, (**A-B**) fixed wing rear spars, (**B-A**) moving wing front spars, and (**B-B**) moving wing rear spars.

**Figure 19:**
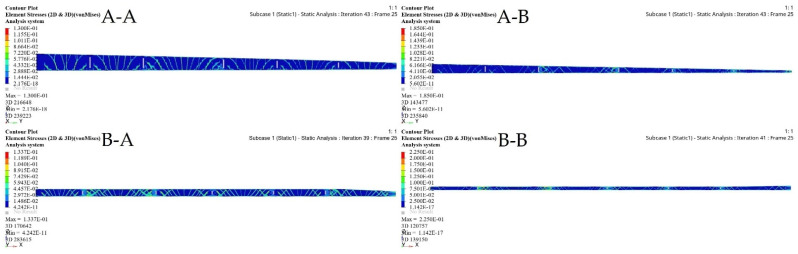
Stress results based on topology optimization for: (**A-A**) fixed wing front spars, (**A-B**) fixed wing rear spars, (**B-A**) moving wing front spars, and (**B-B**) moving wing rear spars.

The merit of TO within OptiStruct is that the FE properties were updated depending on the optimization results. Figure 18 and Figure 19 illustrate the FE characteristics obtained with TO, and Table 5 shows the weights of the initial spars and those of the optimized spars, which reveals the significant reduction in the average weight of the wing spars.

This component simulation-based TO approach guides detailed structural design and provides an alternative way to reduce wing spars’ structural weight. Furthermore, Table 5 shows that the proposed component simulation-based TO approach can be an efficient, as well as logical design technique for the continuum design of wing components with high efficacy and reliability.

**Figure 20:**
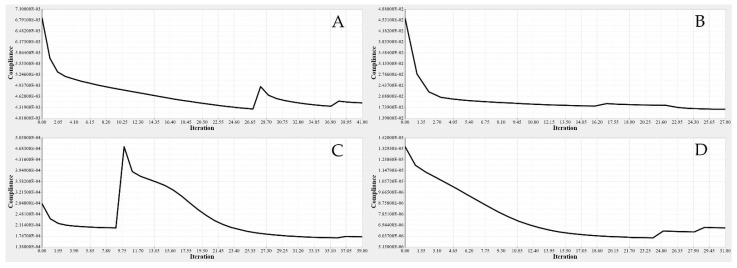
Convergence graphs of the objective functions for: (**A**) rib 1 of a fixed wing, (**B**) rib 2 of a fixed wing, (**C**) rib 1 of a moving wing, and (**D**) rib 6 of a moving wing.

The same procedures applied to the wing spars were chosen for the wing ribs as well. An optimization approach based on the Finite Element Model (FEM) was implemented for each rib, depending on its calculated loading. The material density of each element was defined as a design variable. Two analysis responses expressed in terms of structural compliance and volume fraction were identified, in which the volume fraction was classified as a constraint, and the compliance was defined as an objective function. The typical setup of the TO led to compliance minimization by using the volume fraction parameter as a constraint; the converged solution was obtained using an iterative process, as illustrated in Figure 20.

**Figure 21:**
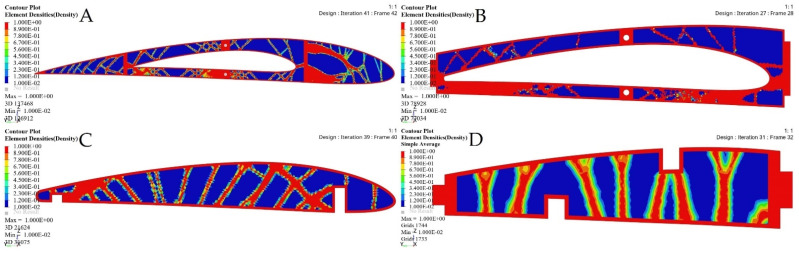
Element density variations with the iteration numbers for: (**A**) rib 1 of a fixed wing, (**B**) rib 2 of a fixed wing, (**C**) rib 1 of a moving wing, and (**D**) rib 6 of a moving wing.

Figure 21 depicts the material density obtained by TO for the wing ribs (for simplicity, Figure 21 shows ribs 1 and 2 for the fixed wing, and ribs 1 and 6 for the moving wing).

**Figure 22:**
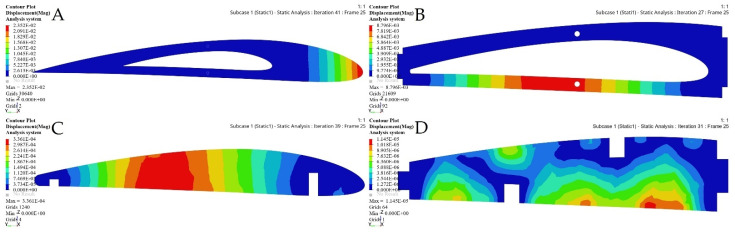
Deformation results based on TO for: (**A**) rib 1 of a fixed wing, (**B**) rib 2 of a fixed wing, (**C**) rib 1 of a moving wing, and (**D**) rib 6 of a moving wing.

**Figure 23:**
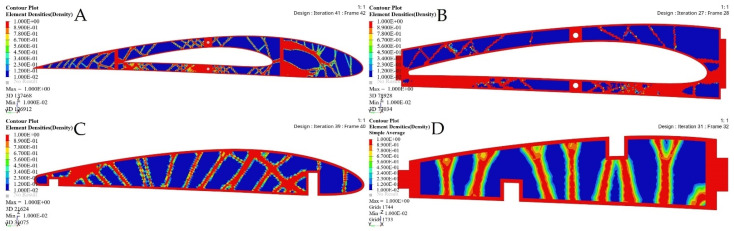
Stress results based on TO for: (**A**) rib 1 of a fixed wing, (**B**) rib 2 of a fixed wing, (**C**) rib 1 of a moving wing, and (**D**) rib 6 of a moving wing.

The FE models based on the TO process for the selected ribs shown in Figure 22 and Figure 23 demonstrate that an important weight reduction was obtained without affecting the components stiffnesses.

Table 6 displays the VSMTW rib TO results obtained using their various starting values based on the aerodynamic loading distribution. The comparison of the weight values of ribs before and after optimization reveals that their weights were substantially reduced following the calculated loads acting on the rib. The maximum stresses and displacements of all ribs were very small; thus, the optimized ribs satisfied the strength and stiffness requirements. The seventh rib of both the fixed and moving wings models were excluded from the optimization process, as these wings must be solid without hollows.

**Figure 24:**
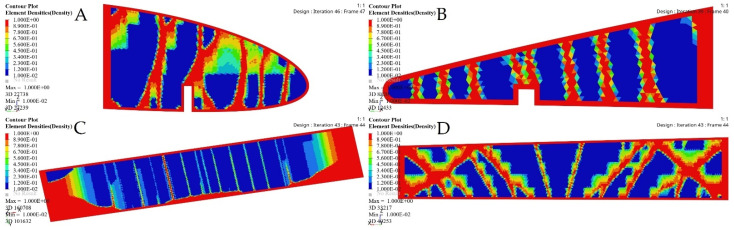
Element density plots with the iteration numbers for fixed wing: (**A**) leading edge of rib 1, (**B**) trailing edge of rib 1, (**C**) bottom support surface between rib 6 and rib 7, and (**D**) front spar support.

**Figure 25:**
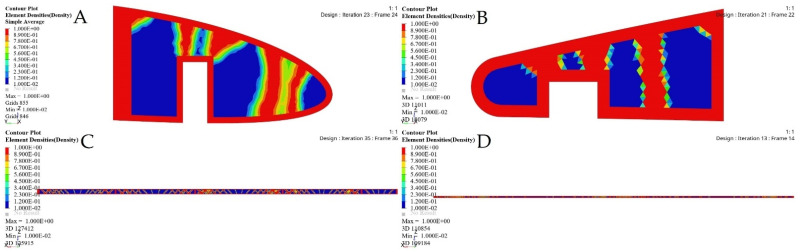
Element density plots with the iteration number for moving wing: (**A**) leading edge of rib 1, (**B**) trailing edge of rib 1, (**C**) support stringer of a front spar, and (**D**) support stringer of a rear spar.

The optimized wing segments modeled as solid suggest that a wing should have strut elements obtained from TO in order to meet their strengths requirements. Various aerodynamic load scenarios obtained for extreme flight conditions were evaluated to design the support parts, as indicated in Table 3, and in Figure 3 and Figure 4. The baseline TO was conducted on the support elements’ structure for both wing segments using the required load conditions. Certain support elements were excluded from the TO due to their geometrical designs. The results obtained from various TOs were represented as density cloud maps for the support elements, as shown in Figure 24 and Figure 25 for fixed wing and moving wing segments, respectively. The red sections represent the solid region, while the blue sections represent the void region.

**Figure 26:**
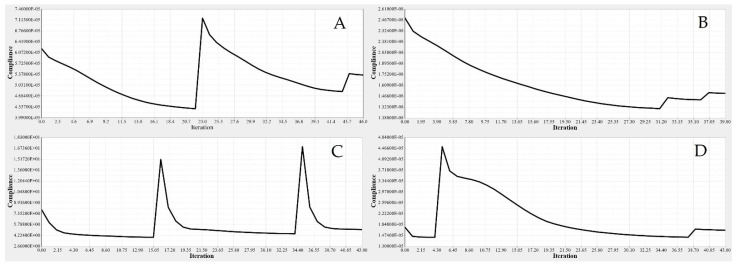
Convergence graphs of the objective function for a fixed wing: (**A**) leading edge of rib 1, (**B**) trailing edge of rib 1, (**C**) bottom support surface between ribs 6 and 7, and (**D**) support of a front spar.

**Figure 27:**
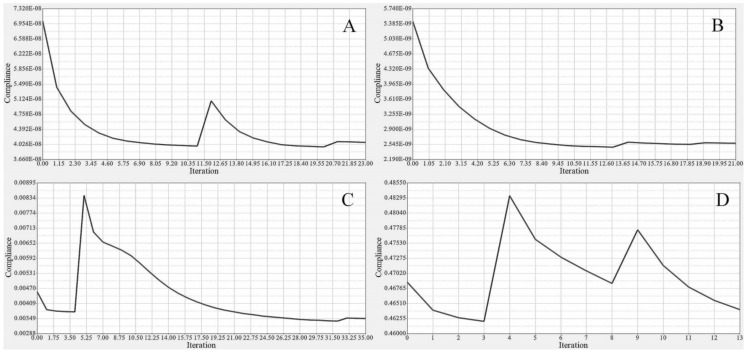
Convergence graphs of the objective function for a moving wing: (**A**) leading edge of rib 1, (**B**) trailing edge of rib 1, (**C**) support stringer of a front spar, and (**D**) support stringer of a rear spar.

The basic structure of support elements was highly minimized based on the TO results, thereby reducing the support elements weights. Moreover, it is clear that the structural compliance decreased as the number of iterations increased, as shown in Figure 26 and Figure 27.

**Figure 28:**
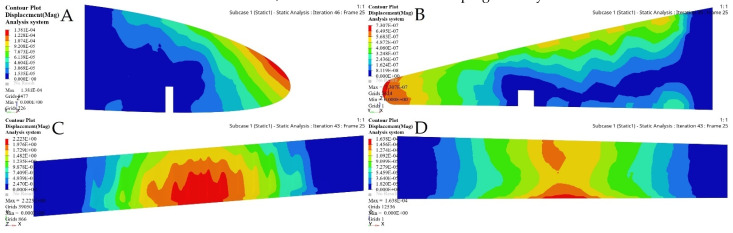
Deformation results based on topology optimization of a fixed wing: (**A**) leading edge of rib 1, (**B**) trailing edge of rib 1, (**C**) bottom support surface between ribs 6 and 7, and (**D**) support of a front spar.

**Figure 29:**
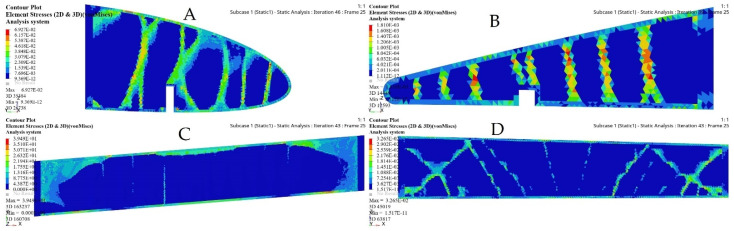
Stress results based on topology optimization of a fixed wing: (**A**) leading edge of rib 1, (**B**) trailing edge of rib 1, (**C**) bottom support surface between ribs 6 and 7, and (**D**) support of a front spar.

**Figure 30:**
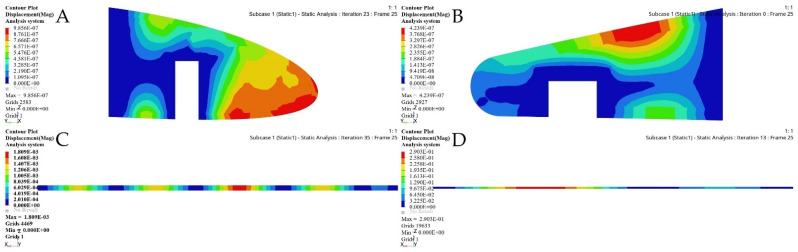
Deformation results based on topology optimization of a moving wing: (**A**) leading edge of rib 1, (**B**) trailing edge of rib 1, (**C**) support stringer of a front spar, and (**D**) support stringer of a rear spar.

**Figure 31:**
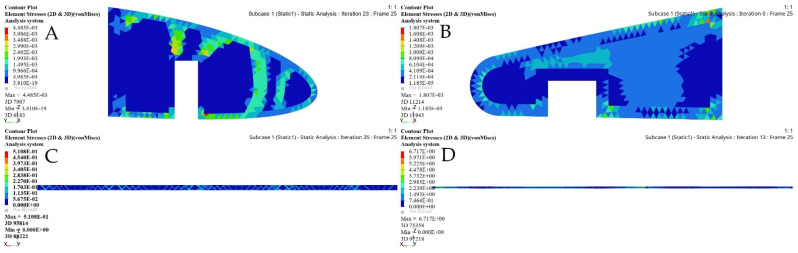
Stress results based on topology optimization of a moving wing: (**A**) leading edge of rib 1, (**B**) trailing edge of rib 1, (**C**) support stringer of a front spar, and (**D**) support stringer of a rear spar.

Figure 28, Figure 29, Figure 30 and Figure 31 show the effects of a TO process on the structural changes in mechanical properties, such as deformations and stresses. These figures indicate that the strain can be reduced, while the structural stiffness progressively increases.

The main parameters’ values were calculated in the optimization analysis. The values of the results depended on the number of iterations and the mechanical analysis and they differed between each individual component of the support elements, as the forces and the geometric shapes differed from one support element to another. Several support elements were eliminated from the optimization process due to the difficulties in their implementation in terms of their geometrical and physical obstacles such as cylindrical shape.

## 7. Remodeling of the Optimized Wing Components and Their Final Design

Generally, the morphing wing configuration is used for the accomplishment of multiple missions in an individual flight. The work presented here was applied on both Hydra Technologies’ UAS-S4 and UAS-S45 Baalam [51]. First, SO was implemented on the wing skin to determine its optimum thickness. Next, TO was utilized to determine the optimum internal wing components that would reduce the components’ weights while maintaining their strengths. Finally, remodeling based on optimization results was considered for the design of wing components’ configurations. This optimization was performed for fixed and moving wing segments, so that the wing component’s structure could be remodeled to further analyze their weight optimization.

The optimized wing model was built from aluminum 2024-T3, with its fixed and moving segments weighing 16.3 kg and 10.3 kg, respectively. The wing components’ structure manufacturing includes the arrangement of the spars, ribs, and support elements based on the TO results, and then, the design of a skeleton model of the wing structure. In the present optimization investigation, the TO method was performed on the wing components, including its spars and ribs. Minor support elements were eliminated from the TO method, as they were ineffective from a structural perspective.

**Figure 32:**
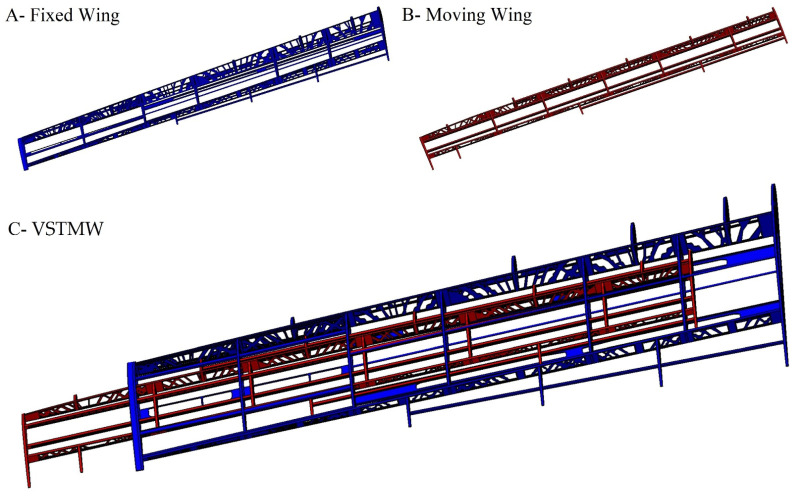
Detailed remodeling of the VSTMW (**A**) fixed wing segment, (**B**) moving wing segment, and (**C**) assembled VSTMW at the original position.

The highest challenge in this phase of optimization was the implementation of the TO on the fixed segment ribs. Given the space required to house the moving segment, we assumed that there should be a cavity in the middle of each rib, as seen in Figure 8. Subsequently, it was decided that reinforcement of the fixed wing’s middle area was needed to support the ribs in the impairment regions where the loads were large, as they resulted from the moving wing segment motion. Both wing segments had to be reinforced due to the shear stress produced by the aerodynamic loads’ reactions under a variety of flight conditions. After remodeling the wing segment shapes based on the TO findings, the CAD model of the design of the MVSTW components was developed according to their density distribution. Figure 32 illustrates the optimized shapes for both wing segments.

After incorporating the structural layout data of the optimized wing components in the whole design, the weights of the optimized wing components were reduced from 16.3 kg for the fixed wing segment and 10.3 kg for the moving wing segment to 7.96 kg and 4.57 kg, respectively.

## 8. Conclusions

This article addresses recent advancements in integrating aerodynamic approaches and optimization methods in order to develop the concept of the morphing variable span of tapered wing (MVSTW) design. This phase of wing component optimization is subordinated to the previous topology optimization phase, which was used to allocate the wing components inside the MVSTW. The optimization was performed using Altair’s OptiStruct solver, connected with the aerodynamic research results obtained using the XFLR5 code. Then, these results were incorporated into SO and TO, and then utilized to solve relevant optimization problems.

The optimization framework for wing components was developed in order to minimize the weights of the MVSTW components while optimizing their structure stiffnesses. The study’s main objective was to identify and thus assess the feasibility of implementing this optimization process for skin and internal wing elements, such as ribs, spars, and others. This novel technique was suggested for the MVSTW based on a telescopic mechanism, which involved sliding a telescopically stretched wing into a fixed wing segment. This approach increased the difficulty of our work by requiring us to pay high attention to wing several components during the optimization process. For example, the fixed wing ribs should have a cavity for sliding the moving segment into the wing to fit a complex geometric shape when applying SO and TO (or STO).

This analysis aims to minimize the weight of the MVSTW by maximizing its stiffness and minimizing its total structural compliance parameters in order to reinforce structural durability, and to satisfy the MVSTW’s structural integrity criteria. The STO findings revealed an advantage in structural weight savings for fixed and moving wing parts components. When the weights of the baseline wing segments were compared to the weights of the optimized wing segments, the optimized wing components’ weights for both segments decreased from 16.3 kg to 7.96 kg for the fixed wing segment, and from 10.3 kg to 4.57 kg for the moving wing segment. Based on the TO results of both MVSTW segments, the wing components’ configurations, including the spars, ribs, and support elements, were redesigned and further developed. In addition, the wing skin was chosen depending on the SO results. The aerodynamic loads were distributed along wingspan length and further obtained under extreme flight conditions. Following the weight savings obtained by the STO techniques on the wing components, the optimized wing shape fulfilled the structural integrity design criteria.

Future research will include additional investigations to develop a reliable adaptive morphing wing. Optimizations will be conducted on composite materials with the aim to achieve greater weight reduction in the MVSTW. Following the optimization of the wing segments using composite materials, the MVSTW configuration and its actuation mechanism integration will be evaluated. The gains obtained by these optimizations will further reduce the fuel consumption.

## Figures and Tables

**Table 1:** Proposed locations of wing ribs for fixed and moving segments; these locations are measured with respect to the reference rib No. 1.

Rib No.	Fixed Wing	Moving Wing
1.	0 mm Reference	0 mm Reference
2.	269 mm	245 mm
3.	534 mm	626 mm
4.	932 mm	919 mm
5.	1198 mm	1234 mm
6.	1503 mm	1502 mm
7.	1800 mm	1875 mm

**Table 2:** Wing aluminum alloy 2024-T3 material properties.

Physical and Mechanical Properties	
Density	2780 kg/m^3^
Ultimate Tensile Strength	483 MPa
Tensile Yield Strength	345 MPa
Modulus of Elasticity	73,100 MPa
Poisson’s Ratio	0.33
Fatigue Strength	138 MPa
Shear Modulus	28,000 MPa
Shear Strength	283 MPa

**Table 3:** Loads calculated for each section of a VSMTW.

Section Number	Lift Load (N)	Ultimate Load
y_1_	257.36	1158.1
y_2_	243.77	1096.97
y_3_	232.15	1044.65
y_4_	213.325	959.96
y_5_	198.29	892.28
y_6_	175.96	791.8
y_7_	158.62	713.77
y_8_	152.7	687.15
y_9_	147.12	662.04
y_10_	134.61	605.75
y_11_	79.11	355.995

**Table 4:** Values obtained from the sizing optimization and Finite Element Analysis for wing skins (a fixed and a moving wing).

	Iterations	Deformation	Stress	Original Skin Thickness	Optimized Skin Thickness	Weight Reduction Ratio
Fixed Wing	4	98.85 mm	200 MPa	2 mm	0.995 mm	52.4%
Moving Wing	3	85.47 mm	199.7MPa	2 mm	0.84mm	58.3%

**Table 5:** Parameters of the wing segments for spars obtained by TO based on Finite Element Analysis.

	Fixed Wing		Moving Wing	
	Front Spar	Rear Spar	Front Spar	Rear Spar
Iterations	43	43	39	41
Initial Weight	1.79 kg	0.82 kg	1.1 kg	0.59 kg
Optimized Weight	0.58 kg	0.37 kg	0.43 kg	0.29 kg
Weight reduction Ratio	67.6%	54.9%	60.9%	50.9%
Deformation	0.00064 mm	0.00117 mm	0.00073 mm	0.00227 mm
Stress	0.13MPa	0.185 MPa	0.134 MPa	0.225 MPa

**Table 6:** Parameters of the wing segments for the ribs obtained from topology optimization based on Finite Element Analysis.

		Iteration	Initial Weight	Optimized Weight	Weight Reduction Ratio	Deformation	Stress
Fixed Wing	Rib 1	41	0.539 kg	0.279 kg	48.2%	0.024 mm	0.85 MPa
	Rib 2	27	0.237 kg	0.1 kg	57.8%	0.0088 mm	2.26 MPa
	Rib 3	26	0.209 kg	0.095 kg	54.5%	0.003 mm	1.11 MPa
	Rib 4	24	0.167 kg	0.09 kg	46.1%	0.023 mm	3.813 MPa
	Rib 5	20	0.137 kg	0.086 kg	37.2%	0.04mm	5.462 MPa
	Rib 6	9	0.107 kg	0.086 kg	19.6%	0.082 mm	5.737 MPa
	Rib 7	0	0.086 kg	0.086 kg	0	0	0
Moving Wing	Rib 1	40	0.134 kg	0.04 kg	70.1%	0.00034 mm	0.19 MPa
	Rib 2	30	0.086 kg	0.028 kg	67.4%	0.00002 mm	0.047 MPa
	Rib 3	30	0.086 kg	0.028 kg	67.4%	0.00002 mm	0.047 MPa
	Rib 4	29	0.086 kg	0.028 kg	67.4%	0.00002 mm	0.045 MPa
	Rib 5	32	0.086 kg	0.028 kg	67.4%	0.00001 mm	0.032 MPa
	Rib 6	31	0.086 kg	0.028 kg	67.4%	0.00001 mm	0.027 MPa
	Rib 7	0	0.113 kg	0.113 kg	0	0	0

## Data Availability

The data presented in this study are available on request from the corresponding author.

## Nomenclature

CAE	Computer-Aided Engineering
CFD	Computational Fluid Dynamics
*E*(*xi*)	Young’s modulus of each element
*Esolid*	Young’s modulus of solid regions
*Evoid*	Very low Young’s modulus
*f*	Distributed body force
*F*	Force vector
FAA	Federal Aviation Regulations
FEM	Finite Element Method
*Fi*	Point load on the *ith* node
*K*	Global stiffness matrix
*k*_0_	Elemental stiffness matrix
MVSTW	morphing variable span of tapered wing
*S*	Surface area of the continuum
SO	sizing optimization
STO	sizing and topology optimization
SIMP	Solid Isotropic Material with the Penalization
*t*	Traction force
TO	topology optimization
*u*	Displacement area
*U*	Global displacement
*U_i_*	ith displacement degree of freedom
*V*	Total volume
*V*_0_	Initial volume
*V_i_*	Volume of the *ith* element
Γ_(*y*)_	Circulation distribution
*X_i_*	Pseudo density
*p*_0_	Density of the base material
*p_i_*	Density of the *ith* element
